# A Study on the Measurement Method of Biot Coefficient for Concrete Based on Experimental Approaches

**DOI:** 10.3390/ma17235868

**Published:** 2024-11-29

**Authors:** Yintao Hu, Nan Ru, Qiujing Zhou, Heng Cheng, Guoxin Zhang

**Affiliations:** 1 China Institute of Water Resources and Hydropower Research, Beijing 100038, China; huyintao@edu.iwhr.com (Y.H.); zhanggx@iwhr.com (G.Z.); 2 State Key Laboratory of Simulation and Regulation of Water Cycle in River Basin, Beijing 100038, China; 3 Science and Technology Promotion Centre, Ministry of Water Resources, Beijing 100038, China

**Keywords:** concrete, Biot coefficient, experiment, pore water, elastic modulus

## Abstract

Concrete stress is a key factor influencing the operational safety of concrete dams, and understanding the true distribution and variation of stress is a major research focus in the field of dam engineering. In the heel region of the dam, internal voids in the concrete may allow external water infiltration under high hydraulic head, leading to changes in the concrete’s elastic modulus and Biot coefficient. These changes, in turn, affect the effective stress experienced by the concrete. Consequently, the measured stress in the heel and toe regions may differ from conventional understanding and standard calculation methods for dam stresses. This is particularly evident in the following aspects: after water impoundment, compressive stress in the dam heel is higher than in the dam toe, with the heel stress exceeding the calculated value by a significant margin, and the variation in stress during the impoundment process being smaller than the calculated value. To address these issues, this paper proposes a theoretical method for measuring the Biot coefficient of concrete through experimental testing and innovatively develops the corresponding experimental equipment. This equipment can accurately simulate the conditions of the dam under different water depths (confining pressures) and measure the deformation of concrete caused by changes in water depth. Based on this equipment, tests were conducted on the elastic modulus and Biot coefficient of dry and saturated concrete specimens under different confining pressures. The Voigt–Reuss–Hill mixed average modulus formula was applied to calculate the elastic modulus of the concrete matrix, exploring the influence of pore water on the mechanical properties of the concrete. The results indicate that the pore water inside the concrete increases its equivalent elastic modulus during the testing process. In numerical simulations of the dam, this increased modulus due to pore water is often overlooked, leading to an underestimation of the results. This partially explains why the measured compressive stress in the dam heel consistently exceeds the calculated values. According to the Biot coefficient calculation theory proposed in this paper, the Biot coefficient of concrete varies with its water content. The Biot coefficient is lower in specimens with high water content compared to those with low water content. Using the Voigt–Reuss–Hill mixed average modulus formula, the elastic modulus of the concrete matrix obtained from the tests was found to be 28 GPa, which is in good agreement with the results from regression analysis. These findings are of significant importance for the safe operation of concrete dam engineering.

## 1. Introduction

Hydraulic structures extensively utilize concrete materials, and the properties of these materials are crucial for the safety and normal operation of dams. Currently, there is a well-established understanding of the influence of pore water on the elastic modulus, compressive strength, and shear strength of concrete [1,2,3,4,5,6]. As dam construction technology has advanced, several world-class ultra-high arch dams have been built in China, such as the Xiaowan, Jinping I [7], Wudongde [8], and Baihetan [9]. However, as the water level rises, the compressive stress at the dam heel remains relatively constant, contradicting the expected tensile stress generated at the dam heel after water storage. The dam heel area is subject to the prolonged exposure of varying high water levels, resulting in different degrees of saturation in the internal pores of the concrete. Consequently, scholars have increasingly focused on the impact of pore water on the safety of operating dams, specifically how pore water influences the stress, strain, and key mechanical parameters of concrete, with the Biot coefficient commonly used to quantify the effects of pore water [10]. The internal voids in the concrete at the dam heel may allow external water infiltration under high hydraulic head, leading to changes in the microscopic mechanical properties of the concrete, such as the elastic modulus and Biot coefficient. These changes, in turn, manifest macroscopically by affecting the effective stress experienced by the concrete. As a result, the measured stress in the heel and toe regions may differ from the conventional understanding and standard calculation methods for dam stress.

In 1923, Terzaghi [11] first introduced the principle of effective stress in soil mechanics, distinguishing it from other mechanics, and derived a model for one-dimensional consolidation problems in soils and pores. In 1941, Biot [12,13] extended the one-dimensional consolidation theory to classical three-dimensional consolidation theory and refined the concept of effective stress. Amos Nur [14] provided an expression for the Biot coefficient in the context of effective stress laws for rocks under fluid action. Zhu Bofang [15], while neglecting skeleton deformation and considering only the effect of pore water pressure on pore deformation, derived the relationships between seepage pressure, stress, and strain in concrete under permeating water, arriving at the same effective stress expression found in soil mechanics. For soil, which is a three-phase granular material [16], the relatively large internal pores result in the material’s bulk modulus (K) being much smaller than that of the matrix (uniform and void-free) ($K_{m}$). Zhu Bofang’s assumptions are entirely valid but do not apply to concrete materials. Accurately measuring the Biot coefficient of concrete has become one of the most important challenges in the field of dam engineering.

There have been numerous studies on the measurement of the Biot coefficient worldwide. Biot and Willis [17] first introduced “jacketed” and “unjacketed” tests, pioneering the original definition and expression of the Biot coefficient, with principles similar to those of the drainage method. Subsequently, various methods were proposed by other researchers, which can generally be categorized into two approaches: the direct method for determining the Biot coefficient and the indirect method, which approximates the Biot coefficient by measuring the volume moduli of jacketed and unjacketed test specimens. Currently, the research on the measurement of the Biot coefficient has primarily focused on rock specimens, and there are no relevant studies on how to measure the Biot coefficient for concrete. The Biot coefficient of concrete has been observed in recent years, with values generally ranging from 0.5 to 0.6 [18], but no experimental verification has been conducted.

In response, this paper proposes a theoretical method for experimentally measuring the Biot coefficient of concrete and innovatively develops corresponding experimental equipment. This equipment can accurately simulate the different water depth (confining pressure) conditions of the dam and measure the deformation of concrete caused by changes in water depth. Based on this equipment, an isolated-water test was designed, and experiments were conducted to measure the elastic modulus and Biot coefficient of dry and saturated concrete specimens under varying confining pressures. The Voigt–Reuss–Hill mixed average modulus formula was applied to calculate the elastic modulus of the concrete matrix, and the influence of pore water on the microscopic mechanical properties of the concrete was investigated. The findings of this study are of significant importance to the construction and operation of concrete dams.

## 2. Experimental Overview

### 2.1. Theory of Biot Coefficient Measurement

The constitutive model of porous media mechanics based on Biot theory is as follows:

(1) $$\left\{\begin{array}{l} \varepsilon_{ii}=\frac{1}{E}\left[\sigma_{ii}-\mu \left(\sigma_{jj}+\sigma_{kk}\right)\right]-\alpha \frac{p}{3K}\\ \gamma_{ij}=\frac{2\left(1+\mu \right)}{E}\tau_{ij}\\ \alpha =1-\frac{K}{K_{m}} \end{array}\right.i,j,k=1,2,3$$

where E, μ, and K represent Young’s modulus, Poisson’s ratio, and the bulk modulus of the material as a whole, respectively. $K_{m}$ denotes the bulk modulus of the aggregate material, while p refers to the pore water pressure. $\varepsilon_{ii}$, $\tau_{ij}$, and $\sigma_{ii}$ correspond to the total strain, shear stress, and normal stress, respectively. The parameter α is the Biot coefficient. For granular materials, K is much smaller than $K_{m}$, allowing α to be approximated as 1. However, for porous continuous media, α cannot be approximated in this manner. When considering the volume compressive deformation of saturated materials:

(2) $$\alpha =1-\frac{K}{K_{m}}=1-\frac{C_{m}}{C}$$

where $C_{m}$ represents the compressibility coefficient of the concrete framework particles, and C denotes the volumetric compressibility coefficient of the concrete. Given that $C_{m}=3(1-2\mu_{s})/E_{s}$ and C=3(1−2μ)/E are known, can derive the following from Equation (2):

(3) $$\alpha =1-\frac{K}{K_{m}}=1-\frac{C_{m}}{C}=1-\frac{E(1-2\mu_{S})}{E_{S}(1-2\mu )}$$

where E, $E_{s}$ and $\mu_{s}$ represent Young’s modulus of concrete, Young’s modulus of the framework particles, and Poisson’s ratio, respectively. When considering concrete an isotropic porous medium, the porosity does not influence Poisson’s ratio; therefore, Equation (3) can be simplified to:

(4) $$\alpha =1-\frac{E}{E_{S}}$$

Equation (4) represents the principle for measuring the Biot coefficient presented in this study. For a given concrete matrix, Young’s modulus (Young’s modulus of ideal non-porous concrete) remains constant, while the initial Young’s modulus is uncertain (the elastic modulus referenced hereinafter corresponds to Young’s modulus). Unlike traditional laboratory methods for measuring the elastic modulus, this study employs a pressure chamber and strain gauges embedded within the concrete to measure the elastic modulus and Biot coefficient of the dam concrete under actual confining pressure. Additionally, it analyzes the effects of pore water on the elastic modulus and the stress-strain relationship.

### 2.2. Experimental Equipment

**Figure 1:**
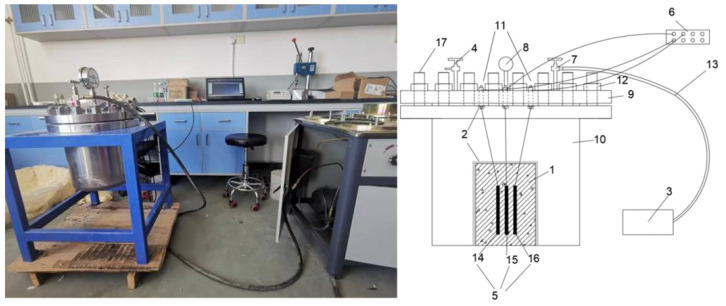
Schematic diagram of the experimental device: (1) Concrete specimen; (2) Seepage control system; (3) Loading system; (4) Unloading system; (5) Measurement system; (6) Data acquisition system; (7) Inlet valve; (8) Pressure gauge; (9) Pressure chamber lid; (10) Pressure chamber body; (11) Terminal block; (12) Nut; (13) Pressure water pipe; (14) Strain sensor; (15) Temperature sensor; (16) Pore pressure sensor; (17) Stud.

To investigate the effects of pore water pressure, the research team developed an experimental device, as shown in Figure 1. This apparatus can accurately simulate and measure the deformation caused by pore water pressure under various water depths (different confining pressures) in the dam. The system consists of a seepage control device, loading and unloading equipment, and measurement and data acquisition equipment. The pressurization device is a digital automatic pressure-regulating plunger pump, which precisely controls the water pressure loading process, with a confining pressure control accuracy of up to 0.01 MPa. The unloading device includes a pressure relief valve capable of quickly releasing the pressure from the specimen and the pressure chamber. The measurement equipment employs embedded vibrating wire strain gauges, which can simultaneously measure the temperature of the specimen. The data acquisition system consists of an eight-channel vibrating wire data logger, with input and output connected to the strain gauges and a PC, respectively. The seepage prevention is achieved using epoxy resin and silicone rubber, while the water isolation is provided by a high-toughness, thickened rubber sleeve.

**Figure 2:**
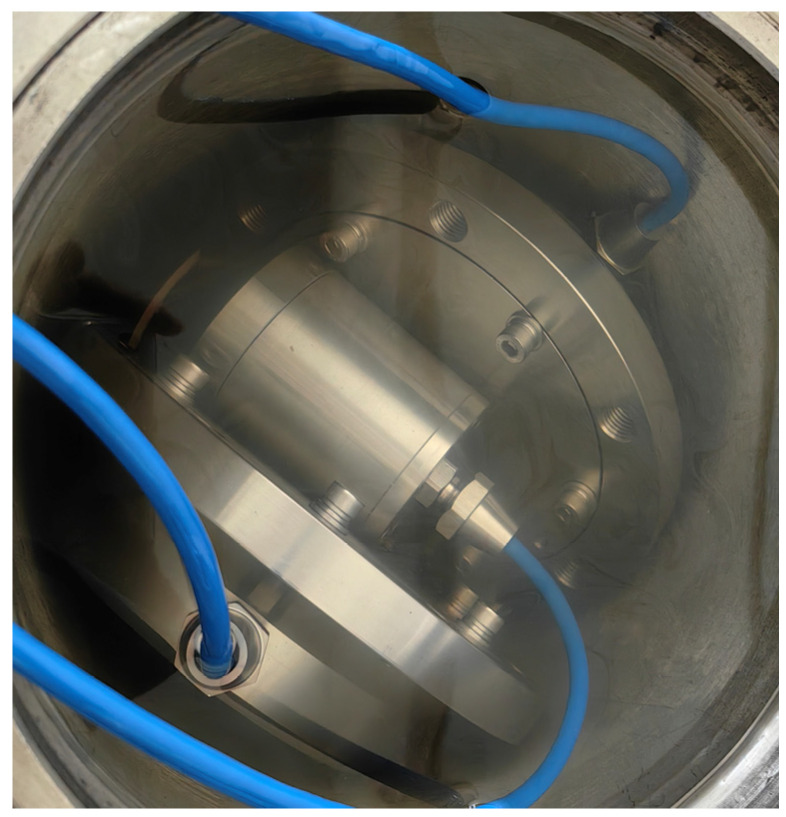
Test of the experimental equipment.

**Figure 3:**
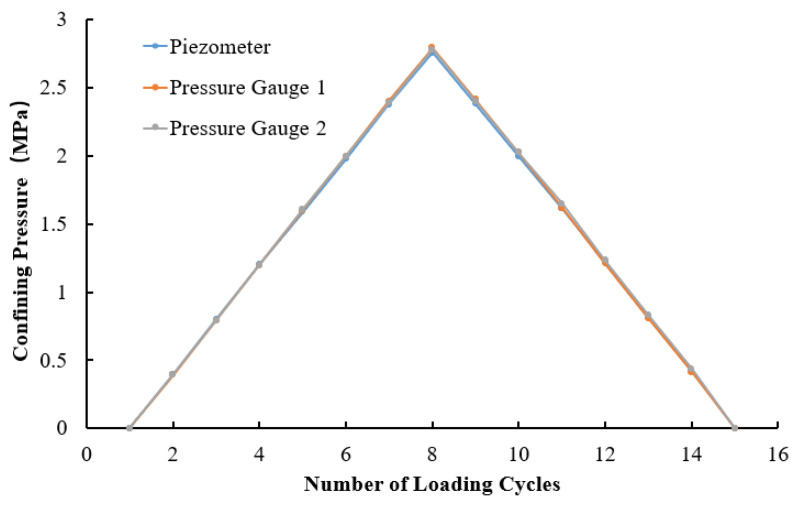
Test results of the experimental equipment.

To ensure measurement accuracy, a preliminary test was conducted to verify the reliability and precision of the experimental setup. The testing procedure is as follows: two calibrated pressure gauges and one calibrated permeation pressure gauge were placed in the pressure vessel in different orientations, as shown in Figure 2. Starting from 0 MPa, pressure was incrementally applied up to 2.8 MPa, with each step increasing by 0.4 MPa, followed by depressurization back to 0 MPa. The test results are shown in Figure 3 and Table 1.

Based on the test results, it can be observed that the measurements obtained from the three instruments during the pressurization and depressurization processes are in good agreement. The error between the actual confining pressure applied by the experimental setup and the expected confining pressure is minimal, meeting the anticipated requirements. This indicates that the experimental setup exhibits high reliability and is suitable for conducting formal experiments.

### 2.3. Experimental Specimens

**Figure 4:**
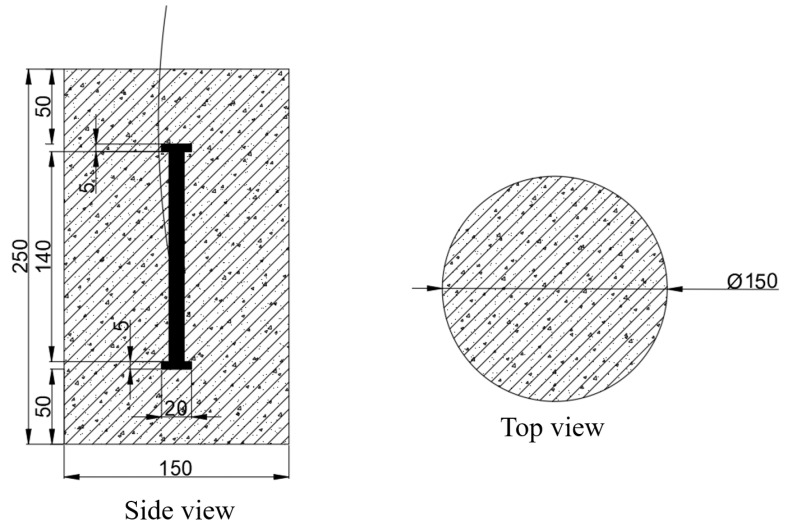
Sample pouring and instrument layout. (Unit: mm).

Two specimens were cast for comparison. The dimensions of the specimens are shown in Figure 4, with a base diameter of 150 mm and a height of 250 mm. The cement used was 42.5 ordinary Portland cement (P·MH 42.5). The concrete mix ratio was: water:cement:sand:gravel = 146:271.1:146:706:1056, with granite as the aggregate and a maximum particle size of 20 mm. An embedded vibrating wire strain gauge was fixed vertically at the center of the mold. After casting, the specimens were placed on a vibration table for adequate compaction. Upon completion, they were first placed in the mixing area and then demolded after final setting, followed by storage in a curing room for 23 days. Finally, they were transferred to a dry shrinkage curing room for an additional 5 days, maintaining an environmental humidity of 60%.

### 2.4. Water Content States of Concrete Pores

**Figure 5:**
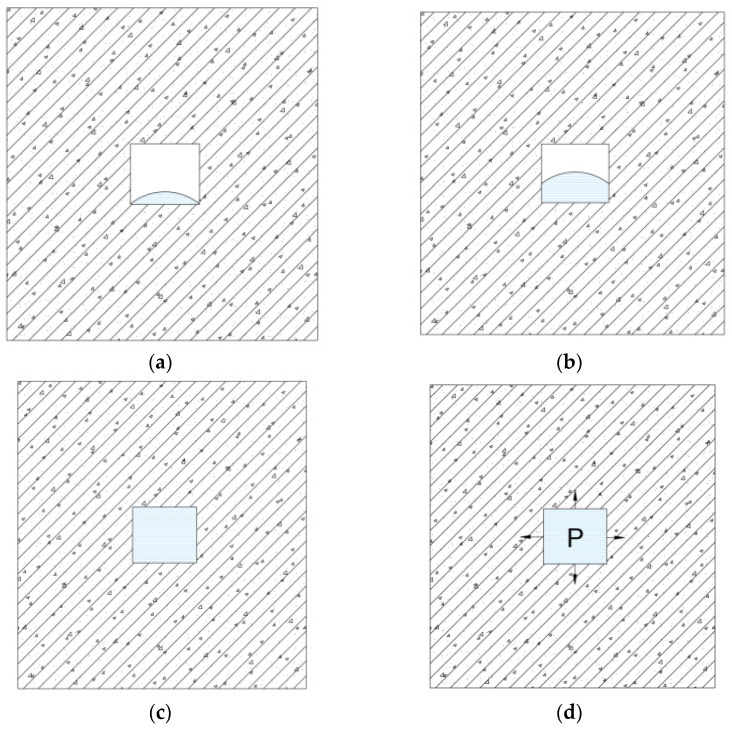
Water content state of concrete: (**a**) Unsaturated state 1; (**b**) Unsaturated state 2; (**c**) Saturated unconfined state; (**d**) Saturated confined state. (P represents the pore water pressure).

Based on the amount of water present within the pore spaces of concrete, the concrete can be classified into three states during testing: (1) Unsaturated state: The pore spaces contain free water but are not completely filled. Under external confining pressure, there is no reactive force acting on the skeleton, as illustrated in Figure 5a,b; (2) Saturated unconfined state (saturated state): The pore spaces are fully filled with free water, and in the absence of external loads, there is no water pressure acting on the skeleton, as shown in Figure 5c, with no pressure exerted on the pore spaces; (3) Saturated confined state (confined saturated state): The volume of free water within the pores exceeds the pore volume, exerting significant pressure on the skeleton. When the external confining pressure is constant, the pore water pressure increases continuously as water infiltrates the pores, eventually stabilizing to a point where the pore water pressure equals the external confining pressure, achieving dynamic equilibrium. However, due to the smaller surface area of the internal pore water pressure compared to the external confining pressure, it still manifests macroscopically as a compressive state, as depicted in Figure 5d.

### 2.5. Theoretical Calculation of Axial Strain Under Confining Pressure

**Figure 6:**
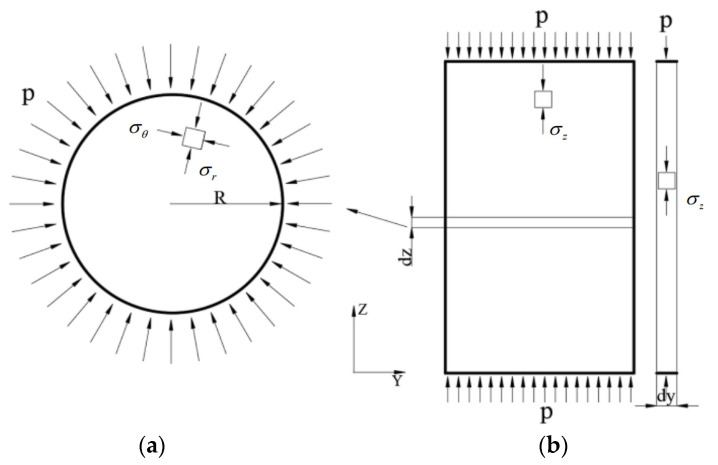
Sample pouring and instrument layout: (**a**) Arbitrary circular cross-section; (**b**) Arbitrary vertical cross-section.

The data collected by the embedded strain gauges during the experiment represent the total strain within the gage length. It is necessary to convert this data into axial strain to reflect the changes in the elastic modulus. The cylindrical test specimens under confining pressure can be categorized based on the confining pressure acting on the surface into two types: transverse circular cross-section and vertical rectangular cross-section, as illustrated in Figure 6a,b. The stress conditions for both types of cross-sections are analyzed as follows:

(1) The elastic mechanics method is employed to analyze the uniformly distributed compressive stress P acting on the lateral surface of the arbitrary thickness dz circular cross-section of the specimen depicted in Figure 6a:

The specimen itself is symmetric under confining pressure, and the stress components $\sigma_{r}$ and $\sigma_{r}$ on the cross-section depend only on the radial coordinate r, and are independent of the axial coordinate θ. By introducing the stress function $\varphi \left(r\right)$ from elastic mechanics, the stress components can be expressed as:

(5) $$\left\{\begin{array}{l} \sigma_{r}=\frac{1}{r}\frac{d\varphi }{dr}\\ \sigma_{\theta }=\frac{d^{2}\varphi }{dr^{2}}\\ \tau_{r\theta }=\tau_{\theta r}=0 \end{array}\right.$$

The corresponding governing equation is:

(6) $$\frac{d^{4}\varphi }{dr^{4}}+\frac{2}{r}\frac{d^{3}\varphi }{dr^{3}}-\frac{1}{r^{2}}\frac{d^{2}\varphi }{dr^{2}}+\frac{1}{r^{3}}\frac{d\varphi }{dr}=0$$

By means of substitution, this Euler equation is transformed into a linear ordinary differential equation, and its general solution is expressed as:

(7) $$\alpha =1-\frac{K}{K_{m}}=1-\frac{C_{m}}{C}=1-\frac{E(1-2\mu_{S})}{E_{S}(1-2\mu )}$$

where A, B, and C are undetermined coefficients, which can be obtained from Equations (5) and (7):

(8) $$\sigma_{r}=\frac{A}{r^{2}}+B\left(1+2Inr\right)+2C$$

(9) $$\sigma_{\theta }=-\frac{A}{r^{2}}+B\left(3+2Inr\right)+2C$$

(10) $$\tau_{r\theta }=\tau_{\theta r}=0$$

The boundary conditions are as follows:

(11) $$\left\{\begin{array}{l} u_{\theta }\to 0:u_{\theta }=\frac{4Br\theta }{E}=0\\ r\to 0,\sigma_{r}\neq 0;r\to R,\sigma_{r}=\left(-\right)p \end{array}\right.\overset{Solve}{\to }\left\{\begin{array}{l} A=0\\ B=0\\ C=-\frac{p}{2} \end{array}\right.$$

Substituting Equation (11) into Equations (8) and (9) yields the stress components at any point in the circular cross-section:

(12) $$\left\{\begin{array}{l} \sigma_{r}=\left(-\right)p\\ \sigma_{\theta }=\left(-\right)p \end{array}\right.$$

(2) Performing a force analysis on the top of an arbitrary vertical rectangular cross-section with thickness dx of the specimen shown in Figure 6b under uniformly distributed compressive stress. By symmetry, it can be inferred that the stress components at any point in the rectangular cross-section are given by:

(13) $$\sigma_{z}=\left(-\right)p$$

**Figure 7:**
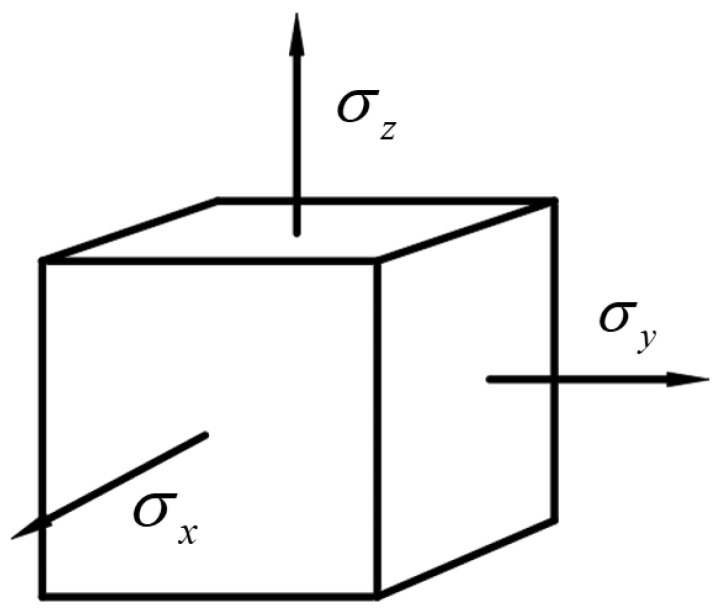
Force acting on an arbitrary infinitesimal element.

(3) Analyzing an infinitesimal element dx, dy, dz at an arbitrary point of the specimen, as shown in Figure 7:

From the previous analysis, it can be concluded that $\sigma_{x}=\sigma_{y}=\sigma_{z}=\left(-p\right)$. According to the generalized Hooke’s Law:

(14) $$\varepsilon_{x}=\frac{1}{E}\left[\sigma_{x}-\mu \left(\sigma_{y}+\sigma_{z}\right)\right]$$

With Equation (14), it can obtained:

(15) $$\varepsilon_{x}=\frac{\sigma_{x}}{E}\left(1-2\mu \right)=0.666\varepsilon$$

where ν=0.167 represents Poisson’s ratio for concrete and ε denotes the axial strain in the X direction. It can be concluded that under the application of confining pressure, the total strain measured by the strain gauge in the X direction, $\varepsilon_{x}$, is approximately 0.666 times the axial strain, ε.

## 3. Water-Pressure Resistance Test of Concrete

### 3.1. Test Conditions

**Figure 8:**
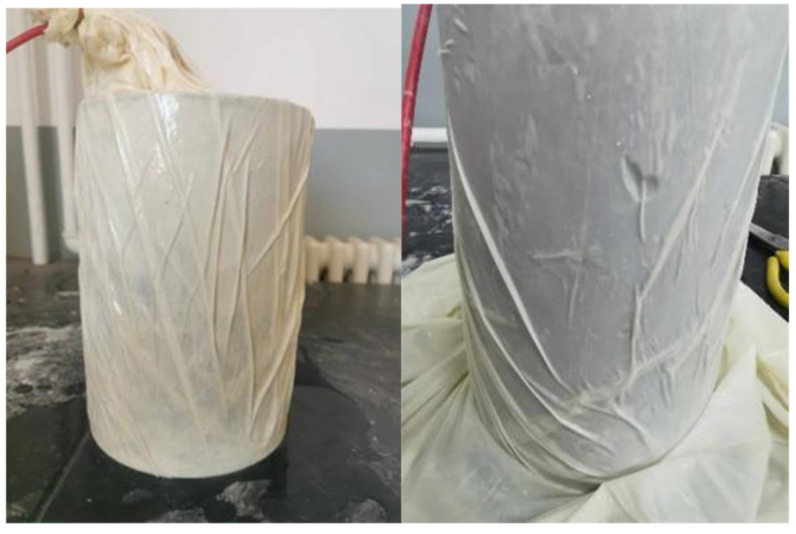
Water isolation treatment of specimens.

The experimental conditions are outlined in Table 2: Each condition begins with a confining pressure of 0 MPa, which is gradually increased in ten stages, with each stage incrementing by 0.2 MPa and maintained for approximately 5 min. After reaching a confining pressure of 2 MPa, the pressure is released back to 0 MPa. The specimen is sealed and water-isolated using multiple layers of high-toughness, thickened rubber sleeves, as shown in Figure 8.

In the experiment, the following assumptions are made regarding the moisture state of the concrete specimens: concrete in an initially dry state is not water-isolated and is subjected to confining pressure within a pressure chamber. Once the strain gauge indicates that the deformation curve stabilizes for a period, it is assumed that the concrete is in a saturated and fully pressured state at that moment. After achieving full pressure saturation, the confining pressure in the pressure chamber is released, allowing the excess water within the concrete to gradually exit the pores under internal pressure. Once the strain gauge indicates that the deformation curve stabilizes again, it is assumed that the concrete is in a saturated and unpressurized state.

### 3.2. Analysis of Experimental Results

**Figure 9:**
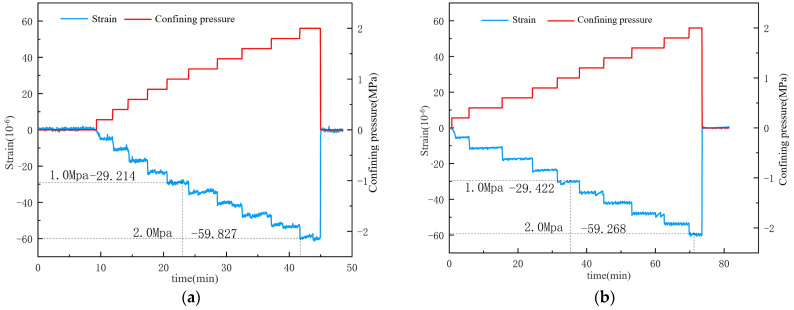
Relationship between strain and confining pressure for dry specimens under condition 1: (**a**) Specimen Number 1; (**b**) Specimen Number.

**Figure 10:**
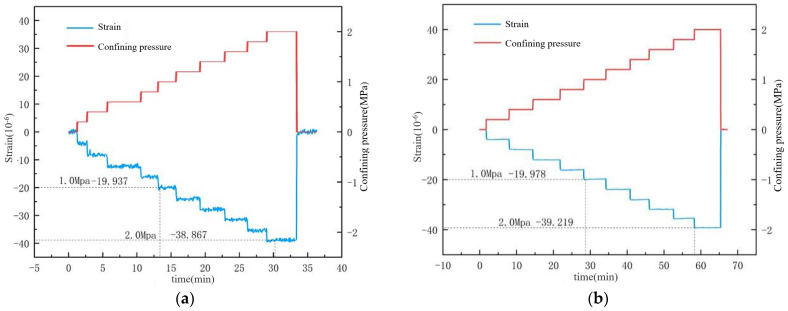
Relationship between strain and confining pressure for saturated specimens under condition 2: (**a**) Specimen Number 1; (**b**) Specimen Number.

After the experiment, the surface water isolation was removed, revealing that the inner layer adjacent to the concrete was dry, indicating effective water isolation and reliable experimental data. As shown in Figure 9 and Figure 10, under water isolation conditions, the deformation patterns of specimens 1 and 2 were consistent, demonstrating high reproducibility. For every 0.2 MPa increase in confining pressure, the axial strain of the unsaturated specimens increased by approximately 6, whereas the axial strain of the saturated specimens only increased by 4 με. Under confining pressures of 1 MPa and 2 MPa, the unsaturated specimens exhibited deformations of approximately 29 με and 60 με, respectively, while the saturated specimens showed strains of only 19.9 με and 39 με. During the sustained confining pressure phase, the experimental loading frequency was rapid, corresponding to dynamic loading. The confining pressure acted on the surface of the specimens, leading to stable strain curves similar to those observed in uniaxial compression tests. Upon removal of the confining pressure, the deformation of each condition returned to zero, indicating effective sealing and satisfactory experimental outcomes.

**Figure 11:**
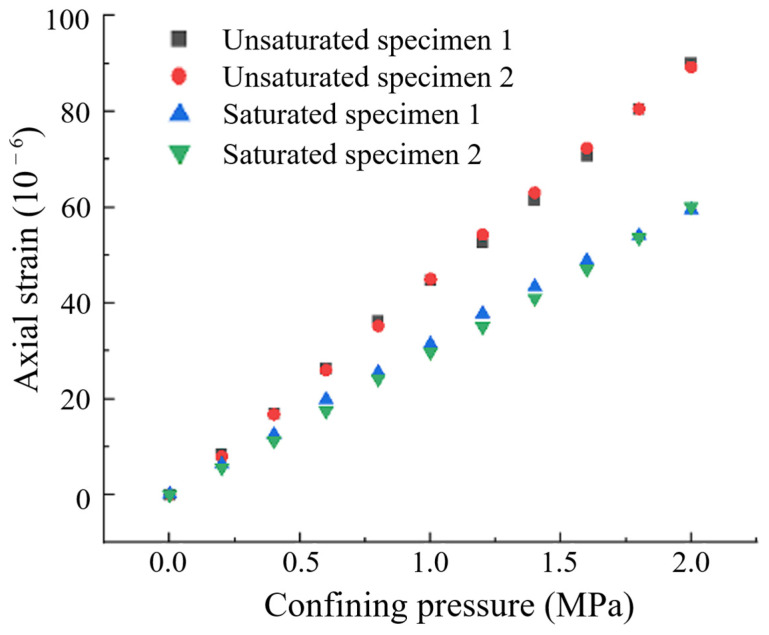
Relationship between strain and confining pressure.

**Figure 12:**
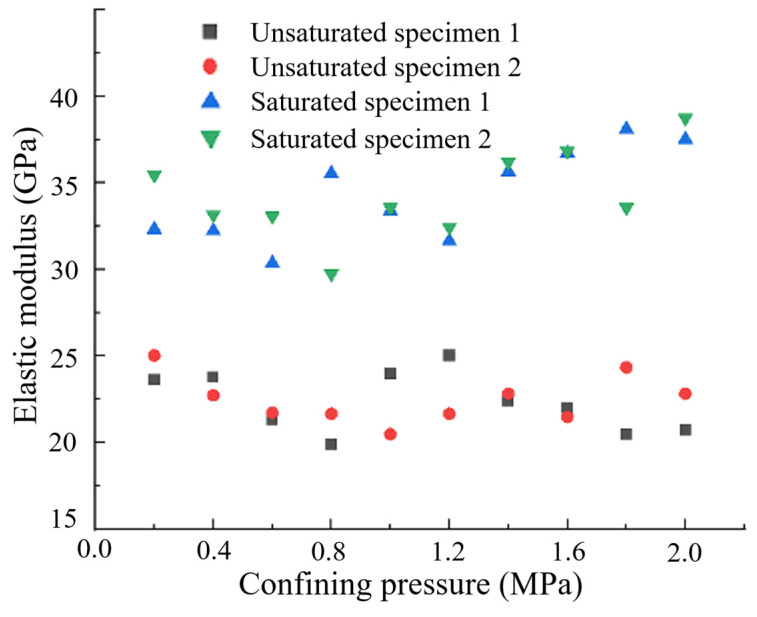
Measured tangent modulus of elasticity under various conditions.

By analyzing the data and applying Equation (15), the measured total strain values were converted to axial strain, as presented in Table 3 and Figure 11. It can be observed that the axial strain of the specimens exhibited a roughly linear relationship with the confining pressure, with consistent trends observed in specimens 1 and 2, demonstrating high confidence in the experimental results. After saturation, the compressive strains generated under the same confining pressure for the same specimen were consistently lower than those in the dry condition. By examining the increments of axial strain associated with changes in confining pressure, the tangent modulus of elasticity of the concrete at each pressurization moment was calculated, as shown in Figure 12. The arithmetic mean of the tangent modulus of elasticity was taken as the elastic modulus of the specimens, with results shown in Table 4. The elastic moduli for the unsaturated specimens 1 and 2 were 22.3 GPa and 22.5 GPa, respectively, while the elastic moduli for the saturated specimens 1 and 2 were 34.1 GPa and 34.3 GPa, respectively. The elastic modulus increased significantly—by nearly 50%—after saturation. This increase is attributed to the high porosity design of the specimens, which facilitates high-pressure water penetration and shortens the experimental duration.

In conclusion, under water isolation conditions, the elastic modulus of the concrete specimens after saturation is significantly greater than that of the unsaturated condition. This can be explained by pore theory: when external free water fills the active voids within the concrete, the contact area between particles increases, leading to greater internal viscous forces and internal frictional forces, thereby enhancing the overall strength and stability of the concrete.

### 3.3. Calculation of the Biot Coefficient

If the porous skeletal material is homogeneous and composed of a single mineral, the bulk modulus $K_{s}$ is equal to the bulk modulus of that mineral. Conversely, if the porous skeleton is heterogeneous and composed of multiple minerals, $K_{s}$ is equal to the weighted average of the bulk moduli of the constituent minerals [19,20]. For example, consider a solid composed of two components with volume fractions $\varphi_{1}$ and $\varphi_{2}$ and their corresponding bulk moduli $K_{1}$ and $K_{2}$. The weighted average modulus can be calculated using the Voigt–Reuss–Hill mixing average modulus formula.

(1) Voigt average modulus:
  (16) $$K_{V}=\varphi_{1}K_{1}+\varphi_{2}K_{2}$$
(2) Reuss average modulus:
  (17) $$K_{r}=1/(\varphi_{1}/K_{1}+\varphi_{2}/K_{2})$$
(3) Voigt–Reuss–Hill mixing average modulus:
  (18) $$K_{S}=(K_{V}+K_{r})/2$$

The strength of hardened concrete is primarily supported by the coarse aggregates and cement paste, which can be viewed as a composition of two components: the paste and the coarse aggregates. The typical bulk modulus of the paste is K = 20 GPa, while the bulk modulus of the coarse aggregate, specifically the granite diorite used in the experiments, is approximately 70 GPa. Based on the mix ratio of the specimens, the volume fraction yields a final bulk modulus for the concrete matrix of 28 GPa. This value is in good agreement with the matrix bulk modulus of K= 27.91 GPa obtained by Yaman [21] through regression analysis. The relationship between Poisson’s ratio and bulk modulus is as follows:

(19) E=3K(1−2μ)

where E represents the elastic modulus, K denotes the bulk modulus, and μ refers to Poisson’s ratio. Consequently, the elastic modulus of the concrete matrix in this study is calculated to be $E_{s}$ = 56 GPa. The Biot coefficients for the dry and saturated conditions are shown in Table 5. After saturation, the Biot coefficient decreases by approximately 34% compared to the dry condition.

## 4. Conclusions

The pore water within concrete increases its equivalent elastic modulus. In numerical simulation calculations of dams, this increase in elastic modulus due to pore water is often neglected, leading to underestimation of the results. This may partially explain why the measured heel pressure stress in dams is often significantly higher than the calculated values. Pore water alters the micro-mechanical parameters of concrete, which in turn affects its macroscopic mechanical behavior, i.e., the influence of the Biot coefficient of the concrete material itself. Currently, there is considerable debate regarding the Biot coefficient value for concrete. Through experiments, this study primarily provides the following insights:

(1) This paper presents a theoretical method for measuring the Biot coefficient of concrete, along with the development of corresponding experimental measurement equipment. This setup can accurately simulate and measure the deformation caused by pore water pressure under different water depths (various confining pressures) in dam conditions.
(2) The elastic moduli of unsaturated concrete specimens 1 and 2 are 22.3 GPa and 22.5 GPa, respectively, while after saturation, they are 34.1 and 34.3. This indicates a significant increase in elastic modulus, nearly 50%. This increase is attributed to the filling of active voids in the concrete with free water, which enlarges the contact surfaces between particles, resulting in enhanced internal viscous forces and internal frictional forces. Consequently, the overall strength and stability of the concrete improves. This conclusion can provide a preliminary explanation for the observed anomaly where the calculated stress values at the dam heel of certain dams are significantly lower than the measured values. The reason for this discrepancy is that the elastic modulus calculations for concrete did not take into account the influence of pore water and instead relied on the smaller values obtained from laboratory tests on dry concrete.
(3) This study applies the Voigt–Reuss–Hill mixed average modulus formula to calculate the elastic modulus of the concrete matrix, yielding a result of 28, which is in good agreement with the regression analysis results obtained by Yaman. Additionally, using the proposed formula for calculating the Biot coefficient of concrete, the Biot coefficients for dry specimens 1 and 2 are found to be 0.601 and 0.599, respectively. Under saturated conditions, the Biot coefficients for these specimens are 0.392 and 0.387. The Biot coefficient of the same concrete specimen varies under different moisture conditions, with the Biot coefficient being lower at higher moisture content than at lower moisture content. However, under the same moisture condition, the difference in Biot coefficients between specimens is small, indicating that despite the segmented construction of the dam using different casting sections, the Biot coefficients remain consistent under the same saturation level. This is likely due to similar concrete mix ratios, curing conditions, and compaction methods across the different sections.

Leveraging the measurement principle and device proposed in this study, further investigation should be conducted on the influence of pore water pressure changes on the deformation of concrete under high confining pressures. This can provide preliminary insights into the abnormal heel stress observed in high dams. Such research is of significant importance to understanding the operational behavior of dams, evaluating the rationality of existing high dam design and analysis methods, and ensuring the safety and control of dam operations.

## Figures and Tables

**Table 1:** Piezometer and pressure gauge readings under different confining pressures.

Confining Pressure (MPa)	Piezometer (MPa)	Pressure Gauge 1 (MPa)	Pressure Gauge 2 (MPa)
0.0	0.000	0.000	0.000
0.4	0.398	0.394	0.396
0.8	0.800	0.792	0.794
1.2	1.201	1.198	1.198
1.6	1.589	1.601	1.608
2.0	1.975	1.995	1.996
2.4	2.374	2.399	2.386
2.8	2.796	2.791	2.789
2.4	2.389	2.411	2.401
2.0	1.997	2.025	2.023
1.6	1.614	1.617	1.627
1.2	1.219	1.210	1.213
0.8	0.800	0.807	0.812
0.4	0.422	0.411	0.416
0.0	0.000	0.000	0.000

**Table 2:** Finite element calculation parameters.

Name	Condition 1	Condition 2
Saturation	0	1
Specimen Number	1 and 2	1 and 2
Sealing status	sealed	sealed
Concrete state	dry	saturated

**Table 3:** Axial strain data.

Confining Pressure (MPa)	Unsaturated Specimen 1 (με)	Unsaturated Specimen 2 (με)	Saturated Specimen 1 (με)	Saturated Specimen 2 (με)
0	0.00	0.00	0.00	0.00
0.2	8.46	7.99	6.34	5.64
0.4	16.88	16.79	12.54	11.25
0.6	26.25	26.01	19.85	17.50
0.8	36.30	35.24	25.48	24.20
1	44.64	45.00	31.47	29.76
1.2	52.63	54.23	37.79	35.09
1.4	61.56	63.00	43.40	41.04
1.6	70.64	72.31	48.84	47.10
1.8	80.42	80.52	54.09	53.61
2	90.08	89.28	59.42	60.05

**Table 4:** Calculation results of tangential modulus.

Confining Pressure (MPa)	Unsaturated Specimen 1 (GPa)	Unsaturated Specimen 2 (GPa)	Saturated Specimen 1 (GPa)	Saturated Specimen 2 (GPa)
0	0.00	0.00	0.00	0.00
0.2	23.64	25.03	32.32	35.46
0.4	23.77	22.73	32.24	33.17
0.6	21.33	21.70	27.36	33.09
0.8	19.90	21.66	35.55	29.76
1	23.98	20.49	33.39	33.59
1.2	25.03	21.66	31.65	32.44
1.4	22.41	22.82	35.65	36.23
1.6	22.01	21.48	36.73	36.83
1.8	20.46	24.34	38.12	33.59
2	20.71	22.82	37.53	38.76
Avg	22.32	22.47	34.05	34.29

**Table 5:** Calculation results of the Biot Coefficient.

Specimen Number	$\alpha_{dry}$	$\alpha_{sat}$
Specimen Number 1	0.601	0.392
Specimen Number 2	0.599	0.387

## Data Availability

The raw data supporting the conclusions of this article will be made available by the authors on request due to privacy reasons.

## References

[1] Zhu J.-G., Li Z.-L. (2017). Effect of Moisture Content on Elastic Concrete Simply Supported Beam. J. Water Power.

[2] Liu B.-D., Lv W.-J., Li L., Li P.F. (2014). Effect of moisture content on static compressive elasticity modulus of concrete. J. Constr. Build. Mater..

[3] Wang H.-L., Li Q.-B. (2006). Effect of pore water on the compressive strength of wet concrete. J. Eng. Mech..

[4] Wang H.-L., Li Q.-B. (2005). Saturated concrete elastic modulus prediction. J. Tsinghua Univ..

[5] Bai W.-F., Chen J.-Y., Sun S.-N. (2010). Effect of Pore Moisture on the Initial Elastic Modulus of Concrete. J. Dalian Univ. Technol..

[6] Li Q.-B., Chen Z.-F.S., Sun M.-Y., Lv P.-Y. (2007). Effect of water loading on strength of concrete. J. Hydraul. Eng..

[7] Wang J.-M., Zheng J. (2021). Research and Practice on Key Technologies in the Construction of Jinping I Hydropower Station. J. Hydraul. Eng..

[8] Ding J., Zhang C.-H., Shao D., Zhang J.-S. (2020). Long-Term Performance of Low-Heat Cement Concrete for the Wudongde Arch Dam. J. Yangtze River Sci. Res. Inst..

[9] Tan Y.-S., Fan Q.-X., Wang Z.-L., Chen W.-F., Guo Z.-G., Lin E.-D., Lin P., Zhou T.-G., Zhou M.-X. (2021). Intelligent Construction Methods for the Baihetan Super High Arch Dam. J. Tsinghua Univ..

[10] Zhang G.-X. (2017). Study on Numerical Simulation Methods for Analyzing the Effect of Seepage Pressure in Continuous Porous Media on Deformation and Stress. J. Hydraul. Eng..

[11] Terzaghi K.T. (1943). Theoretical Soil Mechanics.

[12] Biot M.A. (1941). General Theory of Three-Dimensional Consolidation. J. Appl. Phys..

[13] Biot M.A. (1956). Theory of propagation of elastic waves in a fluid-saturated porous solid. I. Low-frequency range. J. Acoust. Soc. Am..

[14] Nur A., Byerlee J.D. (1971). An exact effective stress law for elastic deformation of rock with fluids. J. Geophys. Res. Atmos..

[15] Zhu B.-F. (1965). The influence of seepage water on the stress state of heterogeneous gravity dams. J. Hydraul. Eng..

[16] Li G.-X. (2010). 50 Lectures on Geotechnical Engineering: A Casual Talk on Rock Foundations.

[17] Biot M.A. (1957). The elastic coefficients of the theory of consolidation. J. Appl. Mech..

[18] Jin F., Liang T. (2009). The difference in concept of uplift force and its recent progress. J. Hydroelectr. Eng..

[19] Berryman J.G. (1992). Effective stress for transport properties of inhomogeneous porous rock. J. Geophys. Res. Solid Earth.

[20] Mavko G., Mukerji T., Dvorkin J. (2009). The Rock Physics Handbook: Tools for Seismic Analysis of Porous Media.

[21] Yaman I.O., Hearn N., Aktan H.M. (2002). Active and non-active porosity in concrete Part I: Experimental evidence. J. Mater. Struct..

